# Use of monoclonal antibodies against Hendra and Nipah viruses in an antigen capture ELISA

**DOI:** 10.1186/1743-422X-7-115

**Published:** 2010-06-03

**Authors:** Cheng-Feng Chiang, Michael K Lo, Paul A Rota, Christina F Spiropoulou, Pierre E Rollin

**Affiliations:** 1 Special Pathogens Branch, Division of Viral and Rickettsial Diseases, Centers for Disease Control and Prevention, Atlanta, Georgia, USA; 2 Measles, Mumps, Rubella and Herpes Viruses Laboratory Branch, Division of Viral Diseases, Centers for Disease Control and Prevention, Atlanta, Georgia, USA

## Abstract

**Background:**

Outbreaks of Hendra (HeV) and Nipah (NiV) viruses have been reported starting in 1994 and 1998, respectively. Both viruses are capable of causing fatal disease in humans and effecting great economical loss in the livestock industry.

**Results:**

Through screening of hybridomas derived from mice immunized with γ-irradiated Nipah virus, we identified two secreted antibodies; one reactive with the nucleocapsid (N) protein and the other, the phosphoprotein (P) of henipaviruses. Epitope mapping and protein sequence alignments between NiV and HeV suggest the last 14 amino acids of the carboxyl terminus of the N protein is the target of the anti-N antibody. The anti-P antibody recognizes an epitope in the amino-terminal half of P protein. These monoclonal antibodies were used to develop two antigen capture ELISAs, one for virus detection and the other for differentiation between NiV and HeV. The lower limit of detection of the capture assay with both monoclonal antibodies was 400 pfu. The anti-N antibody was used to successfully detect NiV in a lung tissue suspension from an infected pig.

**Conclusion:**

The antigen capture ELISA developed is potentially affordable tool to provide rapid detection and differentiation between the henipaviruses.

## Background

Since their first occurrences in 1994 and 1998 respectively, the Hendra (HeV) and Nipah (NiV) viruses have caused recurrent outbreaks throughout northeastern Australia and southern Asia [1-5]. Fruit bats of the genus *Pteropus *have been identified as the primary reservoirs of these viruses [6-9]. Thoroughbred horses and farmed pigs, respectively, were the intermediate hosts between the bat reservoir and humans in the initial outbreaks [10,11]. Since then, several HeV infections had only occurred in horses and no intermediate host was identified in the subsequent NiV outbreaks in India and Bangladesh [5,12-14].

Four fatalities have been reported in 7 cases of human HeV infections [15]. Human case fatalities in NiV outbreaks varied from 38% in Malaysia up to 92% in Bangladesh [2,10,12,13]. The higher case fatalities in the Bangladesh outbreaks could be attributable to bias in selection of admissible patients and lack of adequate healthcare system [2]. Both HeV and NiV are categorized as Biosafety Level 4 (BSL4) Select Agents by the US National Select Agent Program [16,17].

Because HeV and NiV share unique genetic and antigenic features, a distinct genus *Henipavirus, *was created within the family *Paramyxoviridae *[18-20]. Alignments of NiV and HeV amino acid sequences demonstrate similarities ranging from 92.1% for the nucleocapsid (N) protein to 67.6% for the phosphoprotein (P) [19,21]. The divergence in amino acid sequences between NiV and HeV P proteins suggests that it is a potential candidate antigen for differential detection of NiV and HeV.

Infections by NiV or HeV in humans and animals can be confirmed by serologic tests as well as by detection of viral proteins, viral RNA or by virus isolation [16]. The most commonly used serologic assays are ELISAs using infected cell lysate antigens and the specificity of these IgG and IgM ELISA systems for detecting infection with henipaviruses approaches 95% [16]. Recombinant N protein has been used as an alternative antigen for serological detections of henipaviruses in the absence of a BSL4 facility required to generate NiV or HeV infected cell lysate [16,22-25]. Results from ELISA assays can be confirmed by other serologic tests including plaque reduction neutralization [26,27]. A number of sensitive RT-PCR assays have been described for detection of viral RNA [28,29] and these have been used to support outbreak investigations and research. Viral antigen capture ELISA would also provide a high throughput format at relatively low cost. Such assays could be adapted into bedside or pen-side tests to perform rapid detection of henipaviruses in field or clinical settings [30,31].

In this report, we have taken the first steps to develop antigen capture tests for HeV and NiV by characterizing two monoclonal antibodies against the *Henipavirus *P and N proteins. The 2B10 p4 antibody specifically binds and captures HeV P/V/W proteins. The anti-N antibody 1A11 C1 captures proteins from HeV and both NiV Malaysia and Bangladesh strains with high sensitivities, and was able to detect NiV antigen from a pig lung specimen frozen since the Malaysian NiV outbreak. The advantage of this cost-effective assay is that it enables rapid processing of large numbers of specimens, and it can complement the current diagnostic tools for henipaviruses used both in the field and the laboratory.

## Results

### Specificities of monoclonal antibodies to henipaviruses

**Figure 1:**
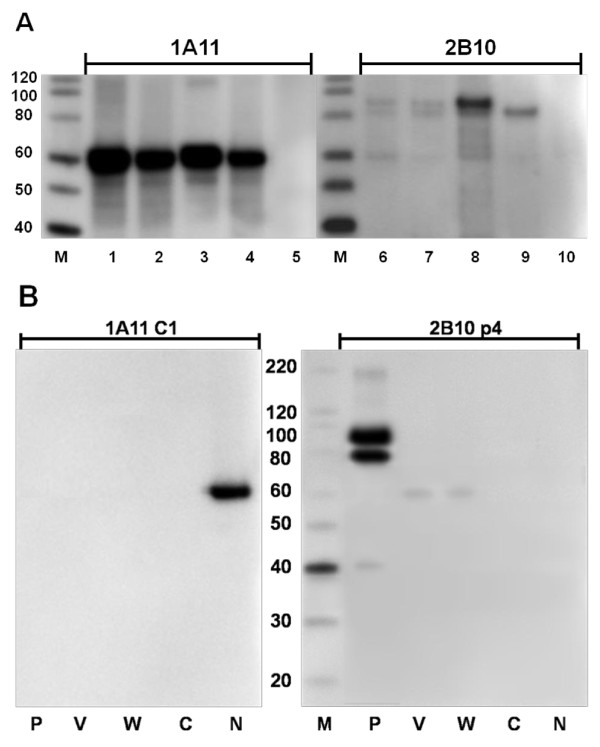
**Characterizations of antibodies produced by hybridomas**. (A) The 4-12% gradient gels were loaded with cell lysate equivalent to 2 μg of protein in each lane as follows, lane 1 and 2 (also lane 6 and 7) represent 2 preparations of NiV Malaysia infected Vero cell lysates; lane 3 and 8, NiV Bangladesh infected Vero cell lysate; lane 4 and 9, HeV infected Vero cell lysate; lane 5 and 10, control Vero cell lysate. After gel separation and transferring, membranes were probed with culture supernatant from 1A11 and 2B10. (B) 293T cells were transfected with NiV Malaysia P, V, W, C or N proteins expressed from plasmids. Ten μL of each cell lysate was separated by SDS-PAGE. Monoclonal antibodies purified from cloned hybridomas were diluted 1 to 2000 and incubated with transferred membranes. Lane M, ladder of MagicMark™ XP Western Protein Standard from Invitrogen.

During the initial rounds of cloning and screening of the hybridomas, two hybridomas (1A11 and 2B10) were selected for their ability to recognize major proteins from HeV and NiV infected Vero cell lysates (Figure 1A). The 1A11 antibody recognized a protein similar in size to the N protein (~58 kDa) from HeV as well as from both strains of NiV (Malaysia and Bangladesh). The 2B10 antibody detected a protein of slightly less than 100 kDa from 2 NiV strains and HeV. It also weakly reacted with a protein similar in size to the N protein in all infected lysates (Figure 1A). These two hybridomas were subjected to further cloning and screening against cell lysates containing individual NiV Malaysia protein P, V, W, C or N, that were expressed from plasmid DNA. The resulting antibody from 1A11 C1 was specific for the N protein (Figure 1B). Antibody from 2B10 p4 strongly recognized the P protein (migrated with apparent molecular weights between 80 and 98 kDa) and a more weakly product of approximately ~40 kDa, which likely represents a degradation or premature termination product. In addition, 2B10 p4 could also weakly detect the V and W proteins (55 kDa, Figure 1B). The C protein which is translated from an alternative reading frame on the P gene was not recognized by either antibody (Figure 1B). Antibody isotypes of 1A11 C1 and 2B10 p4 were determined as IgG2a and IgG1, respectively.

### Epitope mapping of 1A11 C1 and 2B10 p4

**Figure 2:**
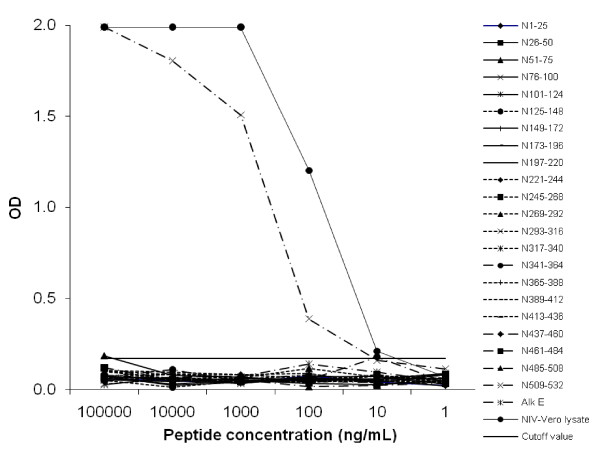
**Epitope mapping of mAb 1A11 C1 using direct ELISA**. Synthetic peptides corresponding to the complete NiV N protein sequence (a.a. 1-532) were serial diluted and coated at concentration from 1 μg to 0.1 ng per well (100 μL in volume). A peptide from Alkhurma virus E protein (Alk E, a.a. 143-168) was included as negative control and signal cutoff value (0.17) was calculated based on readings from this peptide. NiV infected Vero lysate diluted 10 fold to 10^5 ^fold were served as positive control.

**Figure 3:**
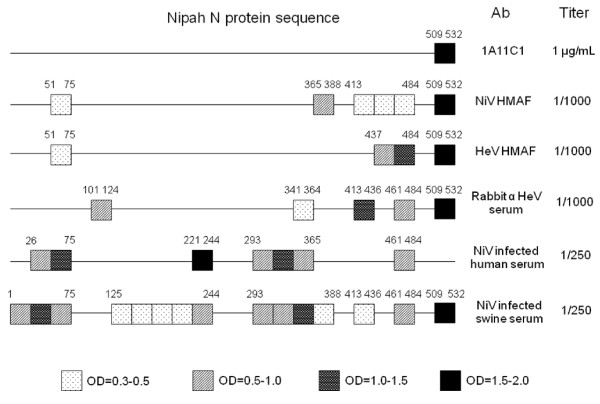
**Diagram of antibody epitopes on NiV N protein sequence**. In addition to the epitope of mAb 1A11 C1, linear epitope mappings were performed with a panel of polyclonal antibodies on NiV N peptides: NiV and HeV hyperimmune mouse ascites fluid (HMAF), Rabbit anti-HeV serum, NiV infected human convalescent serum and a pool of NiV seropositive swine sera. The boxes with illustrated patterns represent the degree of interaction on direct ELISA, and their a.a. positions in N protein sequence were also indicated above.

To determine the linear epitope of monoclonal antibody 1A11 C1, 24 or 25-mers of non-overlapping peptides spanning over the entire N protein sequence of NiV were screened by direct ELISA. Only a C-terminal peptide (a.a. 509-532) yielded specific signals above the 10 ng/ml concentration of plate-coated peptide (Figure 2). The epitope in this C-terminal region was not only detected by NiV hyperimmune mouse ascites fluid (HMAF) but also by HeV HMAF, rabbit anti-HeV serum and a pool of NiV sero-positive swine sera from the 1999 Malaysia outbreak (Figure 3). The overall pattern of shared epitopes among polyclonal antibodies could be observed regardless of the source of immunogen (NiV or HeV) or the mammals from which the antibodies were generated (Figure 3). Interestingly, the rabbit anti-HeV serum bound to a distinct epitope (a.a.101-124) which was not recognized by antibodies generated from other animal species (Figure 3). Several human convalescent sera from the 1999 outbreak did not react to the C terminal peptide of the N protein (Figure 3).

The V and W proteins of henipaviruses are transcribed from the same reading frame as the P protein until reaching the internal mRNA editing site in the P gene [32-34]. We have shown that 2B10 p4 recognized protein products generated from P gene transcripts (NiV P, V, and W proteins) by Western blot (Figure 1B). This suggests that the epitope of 2B10 p4 is located within the common N-terminal sequence (a.a. 1-407) of these proteins. However, linear epitope mapping over this region did not result in any peptide binding even with the inclusion of reported single phosphorylated site at Ser-240 [21] (Data not shown).

### Antigen capture from infected Vero cell lysates

**Figure 4:**
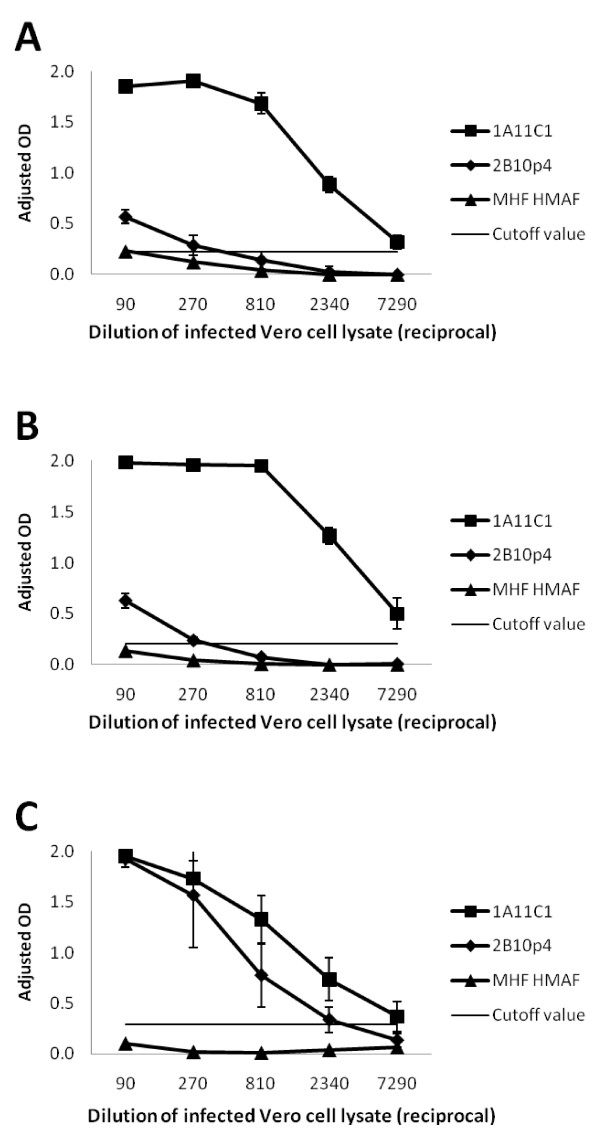
**Antigen capture ELISAs for the detection of NiV or HeV from infected cell lysate**. Serial dilutions of (A) NiV Malaysia prototype, (B) NiV Bangladesh or (C) HeV infected Vero cell lysate was tested with mAbs 1A11 C1 and 2B10 p4 on antigen capture ELISA. Marburg virus (MHF) HMAF was included as negative antibody control. Data points represent the means ± standard deviations from 5 replicates. Signal cutoff values were calculated based on uninfected Vero cell lysate controls.

Monoclonal antibodies 1A11 C1 and 2B10 p4 were further analyzed to test their abilities to capture native viral proteins on ELISA plates. Antibody 1A11 C1 was able to detect NiV Malaysia, NiV Bangladesh and HeV from infected Vero cell lysates (Figure 4A, B and 4C). Successful detection of N proteins was achieved at dilutions ranging 1:1 to 1:7290, with the cutoff value derived from 3 times the standard deviation of the average OD of the uninfected cell lysate controls being 0.2-0.3. The P/V/W specific antibody 2B10 p4 could only detect NiV proteins at very low dilutions (1:1 to 1:270, Figure 4A and 4B); however, applying infected cell lysate at a dilution less than 1:270 resulted in increased non-specific signals (*e.g. *Marburg hemorrhage fever (MHF) virus HMAF coated controls in Figure 4A, B and 4C). The 2B10 p4 captured HeV P/V/W protein at cell lysate dilutions of 1:1 to 1:2340 (Figure 4C).

### Sensitivity of antigen capture ELISA

**Figure 5:**
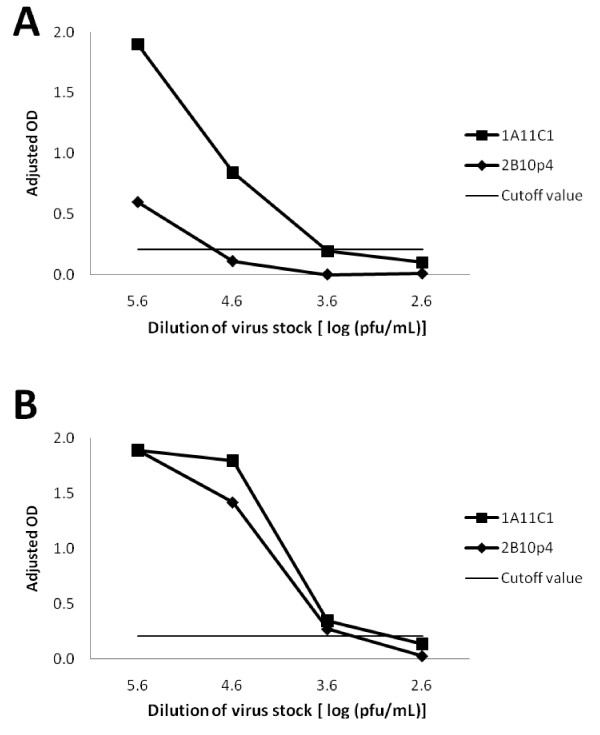
**Sensitivities of antigen capture ELISAs for titrated NiV and HeV stocks**. (A) NiV Malaysia prototype stock (4.1 × 10^6 ^pfu/mL) and (B) HeV stock (1.9 × 10^6 ^pfu/mL) were serial diluted onto wells coated with 1A11 C1 and 2B10 p4. Marburg virus (MHF) HMAF was included as negative antibody control and signals above this antibody control were shown (Adjusted OD). Lassa virus stock (1 × 10^8 ^pfu/mL) was used as negative virus control and signal cutoff value was calculated based on its OD readings.

Serial dilutions of titrated virus stocks (NiV Malaysia prototype and HeV) were prepared in buffer containing nonionic detergent and tested on antigen capture ELISA coated with 1A11 C1 or 2B10 p4 (Figure 5A and 5B). Antibody 1A11 C1 was capable of capturing NiV or HeV at virus titer of log 3.6 pfu/mL (400 pfu per well). The anti-P 2B10 p4 antibody detects HeV at a comparable sensitivity as 1A11 C1 (Figure 5B), but had poor ability to bind NiV (Figure 5A) similar to results obtained with the infected Vero cell lysate (Figure 4A). Dilutions of Lassa virus were included as the background control to calculate the cutoff value of the assay (0.21, Figure 5A and 5B).

### Detection of NiV in pig tissue by antigen capture ELISA

In order to evaluate antigen capture ELISA, cell suspensions from γ-irradiated pig tissue specimens from the Malaysian outbreak in 1999 were prepared as target antigens on plates coated with monoclonal antibody 1A11 C1. The results of the antigen capture assay are shown alongside data from RT-PCR, virus isolation, immunohistochemistry (IHC), and antibody detections [10] in Table 1. A low titer of NiV N antigens was detected in the lung of pig 55 using the antigen capture assay (Table 1). Positive RT-PCR, virus isolation and IHC results also confirmed the existence of NiV in the lung of this pig (Table 1). However, the antigen capture assay did not detect viral antigen in the lung and brain of Pig 5, although RT-PCR and virus isolation performed at the time of the outbreak confirmed NiV infections in the lung of Pig 5 (Table 1). No virus was detected in the brain, lung, or kidney of Pig 4 and 59 by capture ELISA, RT-PCR, virus isolation, or IHC (Table 1). No IgM or IgG response was detected during the time of outbreak in the serum of these pigs (Table 1). Due to the shortage of outbreak specimens, we were unable to determine the sensitivity and specificity of antigen capture ELISA on tissue samples.

**Table 1:** Diagnostic result of NiV infections in pigs^a^.

Pig no	Capture ELISA^b^	RT-PCR^c^	Virus isolation	IHC^c^	Ab^d^
4	-	-	-	-	-
5	-	+	+	ND	-
55	+ (lung, 4)	+	+	+	-
59	-	-	-	-	-

^a^+ or -, samples positive or negative on capture ELISA, RT-PCR, virus isolation, IHC and antibody detection.

^b^Positive identified tissue and the reciprocal titer of 10% tissue suspension in parentheses.

^c^Lung tissue samples were used in RT-PCR and IHC. Primer sequences and procedures were described in [10]. ND, not done.

^d^Both serum IgM and IgG detection assays were performed.

## Discussion

Up until the present, all the reported cases or outbreaks of infection with henipaviruses were within the geographical distribution of *Pteropus *spp. [6,8,9]. These bats appear to settle into subpopulations with limited interactions among colonies [1]. NiV and HeV apparently remain separate within their hosts and respective regions with little overlap [1]. On the other hand, other families of bats were often found coexisting in the same colony with *Pteropus. *Antibodies reactive with Nipah virus were found in *Eidolon dupreanum *in Madagascar [35]. Furthermore, other novel henipavirus-like sequences and cross-reacting antibodies have recently been identified in *Eidolon helvum *from Ghana, West Africa [36,37], which potentially implicates much wider endemic regions of henipaviruses than previously known.

Here we report two monoclonal antibodies that recognized native conformations of N and P/V/W proteins of henipaviruses. In previous studies, monoclonal antibodies were produced either through phage display library screening, or using chemically inactivated virus/recombinant viral protein immunizations [22-26,38-40]. The hybridomas in our study originated from mice immunized with γ-irradiated virus, and the secreted antibodies recognized native viral proteins. Linear epitope mapping on NiV N sequence indicates the epitope of 1A11 C1 is located within the last C-terminal 23 amino acids (a. a.) of the N protein (509-532). Alignment data of the C terminal area of N protein between NiV and HeV further indicated that the last 14 a.a. (520-532) were identical between the two viruses [41], and would likely represent the actual epitope. Interestingly, this epitope is located right after the N-P interaction site (a.a. 468-496) on the N protein [41]. Epitope mapping of other monoclonal antibodies to recombinant N protein or phage library screening of infected swine sera did not identify the C terminus of N protein as a site of antibody recognition [40,42]. In addition, linear epitope mappings against the NiV N protein sequence using ascites fluids, sera from immunized animals or infected humans/pigs were performed and compared. The C terminal peptide of N protein was found to be a strong epitope recognized by all of polyclonal antibodies tested except for NiV infected human convalescent sera.

Our antigen capture ELISA, using plates coated with anti-N 1A11 C1 antibodies was capable of detecting HeV and NiV at a lower limit of detection of 4000 pfu/mL which is comparable to detection sensitivities reported in other antigen detection ELISAs [43,44]. The 1A11 C1 or 2B10 p4 antigen detection assay also demonstrated its specificity by low background signal cutoff values for uninfected Vero cell lysate and Lassa virus. In addition, when using high dilutions of NiV/HeV infected Vero cell lysates (≥ 1: 300), non-specific signals were kept below background levels when polyclonal antibodies against MHF or Crimean-Congo hemorrhagic fever (CCHF) virus (data not shown) were coated on the plate.

Monoclonal antibody 1A11 C1 was also shown to capture NiV from one frozen pig lung specimen from the initial Malaysian NiV outbreak. However, corresponding RT-PCR and virus isolation results obtained at the time of the outbreak suggest the assay failed to identify another infected pig. A previous study had shown virus titers of 5 × 10^7^, 2.5 × 10^3^, and 6.3 × 10^5 ^pfu/g of swine CSF, lung, and spleen tissues, respectively [45]. In our antigen detection assay, 10% tissue suspension (wt/vol) was used as the origin of serial dilutions. Virus loads in some tissue may be too low to be detected at this dilution range. Furthermore, viral degradation resulting from long term storage may further compromise the results of antigen capture ELISA. Unfortunately, we were unable to confirm this possibility by repeating RT-PCR and virus isolation since tissue samples were irradiated.

A previous study has described using monoclonal antibodies against N and M proteins to differentiate NiV from HeV by Western blot [38]. In our study, 2B10 p4 antibody specifically captured native HeV P/V/W proteins and could only detect NiV proteins at high virus concentration or by Western blot. These results suggest that the binding affinity of 2B10 p4 could be influenced by how its epitope was presented by the P proteins of NiV and HeV. In fact, we were unable to identify linear epitope of 2B10 p4 from the NiV Malaysia P sequence despite knowing it should be located within the shared N-terminal sequence of P/V/W protein (a.a. 1-407). In contrast, polyclonal HMAF raised against NiV was able to recognize 6 individual plate-coated peptides in this region by direct ELISA (data not shown).

NiV and HeV soluble recombinant G proteins coated on beads had previously been developed and utilized in a Bio-Plex protein array to differentiate between infections with these viruses [37,46]. As the G proteins of these viruses share 83.3% homology [19] and the assay would be assessing polyclonal antibodies present in clinical specimens, it is unclear to what extent the ability to differentiate between the viruses would be maintained on analysis of diverse specimens from humans, bats or pigs. One of the advantages of the antigen capture assay described here is that it is based on the P protein of NiV and HeV which is highly diverse and share only 67.6% homology [19], which may facilitate the ability to robustly differentiate among infections with henipaviruses.

Real time RT-PCR has been shown to detect Nipah virus with high sensitivity and specificity [28,29]. However, nested RT-PCR using broad-range primers and sequencing may be required to identify newly emerging henipaviruses [36,47]. Although further validation for our antigen capture assay will be needed once HeV and NiV infected tissue specimens are available, this relatively inexpensive and robust diagnostic tool could be useful in broad spectrum surveys for detection of henipaviruses.

## Conclusions

Two monoclonal antibodies were selected to set up antigen capture ELISAs for Henipavirus. Mab 1A11 C1 recognized C terminus of N protein of both NiV and HeV with high sensitivity. 2B10 p4 was found to bind Hendra P/V/W with good specificity. While the applications of NiV/HeV infection by our antigen capture ELISAs remain to be fully evaluated, we believe these assays could offer economical and rapid sample processing for any future outbreaks of henipaviruses.

## Methods

### Virus stocks, cell lysates, and pig tissue suspensions

Hendra virus strain 9409-30-1800 Australia was originated from a horse lung sample from Brisbane, Australia in 1994. Both Nipah virus strain SPB199901924 Malaysia prototype and strain SPB200401617 Bangladesh were isolated from CSF of patients in the 1999 and 2004 outbreaks, respectively. Lassa virus Josiah strain isolated from human in Sierra Leone was used as the control virus stock. Viruses were inoculated in Vero-E6 cell and propagated until CPE reached 3-4+ before harvest. Viruses were titrated by plaque assays on Vero-E6 cells as described [48]. The attached cells were scraped and centrifuged, washed before lysed in borate saline containing 1% Triton X-100, pH 9. The sample was frozen at -70°C and gamma irradiated at 5 × 10^6 ^Rad. The resulting material was sonicated and centrifuged to obtain cell lysate. Control Vero cell lysate was made the same way except no virus infection was performed.

For pig tissue samples from 1999 Malaysia outbreak [10], 10% (wt/vol) suspensions of thawed frozen tissue sections (~250 mg) were homogenized on ice in Hank's balance salt solution (HBSS)/5% fetal calf serum with a plastic pestle and 250 mg of alundum (Fischer Scientific, Pittsburgh, PA) in 15 mL conical tubes. The tissue suspensions were clarified by low speed centrifugation before using in antigen capture ELISA as follows.

### Nipah hybridomas and monoclonal antibodies

Twenty five μL of Nipah virus Malaysian prototype stock was *i.c. *injected into suckling mice in BSL4 laboratory. The brain of the sick suckling mouse was taken out by day 3, γ-irradiated and used for following immunizations. Four weeks old BALB/C mice (Charles River Laboratories, Wilmington, MA) were immunized *i.p. *with 10% suckling mouse brain, HBSS and 0.05 M Tris buffer, pH 9 emulsified with Ribi adjuvant (Ribi ImmunoChem Research Inc., Hamilton, MT) in 200 μL. Two booster injections of 100 μL were given on days 21 and 35. Mice serum antibody titers were monitored by ELISA and IFA in between boosters. One hundred μL of brain homogenate without adjuvant was injected at day 56 and 4 days later, the splenocytes of the immunized mice were isolated and fused with NS-1 (TIB-18) myeloma cells. Hybridomas were screened for secretion for desired antibodies by ELISA and IFA. Western blot was used for confirmations of monoclonality and specificity of the antibody. Protein G 8 mL or Protein A 36 mL column connected to an ÄKTAprime™ plus system (GE Healthcare, Piscataway, NJ) was used for purification of monoclonal antibody from supernatants from hybridoma cultures. Antibodies were concentrated in Amicon 50 mL Stirred Ultrafiltration Cells (Millipore, Billerica, MA). The isotype of purified monoclonal antibody was determined by IsoStrip Mouse Monoclonal Antibody Isotyping Kit (Roche Diagnostics, Indianapolis, IN).

### Western blot

HEK 293T cells were transfected with expression plasmids containing P, V, W, C, or N as previously described [34]. Cells were lysed in RIPA buffer (150 mM NaCl, 1% NP-40, 0.5% sodium deoxycholate, 0.1% SDS, 50 mM Tris Buffer, pH 8). Ten μL of cell lysate was separated on a NuPAGE™ 4-12% Bis-Tris gel (Invitrogen, Carlsbad, CA), then transferred onto a PVDF membrane by iBlot™ Gel Transfer System (Invitrogen). After Blocking with PBS containing 0.05% Tween-20 and 5% skim milk, the membrane was incubated with hybridoma cell supernatant overnight at 4°C. PBS containing 0.05% Tween-20 was used for thorough washing before HRP- conjugated antibody was added to incubation. Signals were developed by using SuperSignal™ West Dura Extended Duration Substrate (Thermo Scientific, Waltham, MA).

### Epitope mapping

Non-overlapped 24, 25 or 26-mer peptides spanning N-terminal half (a.a. 1-407) sequence of Nipah P protein (NCBI Reference Sequence, Accession no: NP_112022) and the entire sequence (a.a. 1-532) of Nipah N protein (NCBI Reference Sequence, Accession no: NP_112021) were synthesized and RP-HPLC purified by the Biotechnology Core Facility in CDC. Peptides were dissolved in water before transferring to microtiter plates, and dried at 37°C overnight as described [49]. The wells were blocked with 100 μL of PBS containing 0.1% Tween-20 and 5% skim milk for 1 hour then washed with PBS containing 0.1% Tween-20. The monoclonal antibody (2B10 p4 or 1A11 C1) or polyclonal antibody (NiV HMAF, HeV HMAF, rabbit anti-HeV serum, NiV infected human convalescent or a pool of NiV seropositive swine sera) was diluted into 1 μg/mL (monoclonal) or 1 to 1000 (polyclonal) with blocking buffer. The antibody was added and incubated for 1 hour at 37°C. The secondary HRP-conjugated goat anti-mouse IgG (Thermo Fisher Scientific, Rockford, IL), goat anti-rabbit IgG (Bio-Rad, Hercules, CA), mouse anti-human IgG Fc (Accurate, Westbury, NY), or goat anti-swine IgG (KPL, Gaithersburg, MD) in 1:8000 was added after washing. After one hour of incubation, color development was measured as described in the following ELISA protocol.

### Antigen capture ELISA

The design and setup of antigen capture ELISA for henipavirus were based on the assay developed for Ebola virus as described previously [43,50]. Five μg/mL of monoclonal antibody (1A11 C1 or 2B10 p4) in 100 μL/well were coated onto each well of microtiter plates (BD Falcon, San Jose, CA) overnight at 4°C. Wells were blocked for an hour at 37°C with PBS containing 0.1% Tween-20 and 5% skim milk then washed with PBS containing 0.1% Tween-20, which also included in subsequent steps. Henipavirus stocks, infected Vero-E6 cell lysate or euthanized pig tissue suspension were serial-diluted with PBS containing 0.1% Tween-20 and 5% skim milk in 100 μL on plate then incubated for one hour at 37°C. Rabbit anti-HeV polyclonal antibody diluted 2000 fold was added and incubated for an hour at 37°C. The wells were incubated with goat anti-rabbit HRP conjugate (Bio-Rad) at a dilution of 1:8000 for 1 hour at 37°C. The peroxidase reaction was developed with ABTS (2, 2'-azino-bis (3-ethylbenzthiazoline-6-sulphonic acid)) substrate system (KPL) for 30 min and optical density (OD) was read at 410 nm (Dynatech MR5000). The OD value subtracted by the background value of control uninfected Vero cell lysate or tissue suspension incubated with coated Marburg virus HMAF (1:1000) was indicated as "Adjusted OD". Test samples were considered positive if their mean OD were greater than the mean OD of uninfected Vero cell lysate or tissue suspension incubated with coated monoclonal antibodies plus 3 times of their standard deviation (indicated as the signal cutoff value).

## Competing interests

The authors declare that they have no competing interests.

## Authors' contributions

CFC performed much of the Nipah and Hendra virus Western blots, ELISAs, and drafted the manuscripts, MKL prepared transfected 293T cell lysates containing NiV proteins and contributed to manuscript preparation, PAR and CFS provided valuable opinions to the results and also contributed to manuscript preparation, PER oversaw the overall assay designs, coordinated study support, and assisted manuscript preparation and submission. All authors have read and approved the final version of this manuscript.
